# ZBTB7A prevents RUNX1-RUNX1T1-dependent clonal expansion of human hematopoietic stem and progenitor cells

**DOI:** 10.1038/s41388-020-1209-4

**Published:** 2020-03-02

**Authors:** Enric Redondo Monte, Anja Wilding, Georg Leubolt, Paul Kerbs, Johannes W. Bagnoli, Luise Hartmann, Wolfgang Hiddemann, Linping Chen-Wichmann, Stefan Krebs, Helmut Blum, Monica Cusan, Binje Vick, Irmela Jeremias, Wolfgang Enard, Sebastian Theurich, Christian Wichmann, Philipp A. Greif

**Affiliations:** 1 Department of Medicine III, University Hospital, LMU Munich, 81377 Munich, Germany; 2 German Cancer Consortium (DKTK), Partner Site Munich, 81377 Munich, Germany; 3 German Cancer Research Center (DKFZ), 69121 Heidelberg, Germany; 4 Anthropology & Human Genomics, Department of Biology II, LMU Munich, 82152 Martinsried, Germany; 5 Department of Transfusion Medicine, Cell Therapeutics and Hemostasis, University Hospital, LMU Munich, 81377 Munich, Germany; 6 Gene Center–Laboratory for Functional Genome Analysis, LMU Munich, 81377 Munich, Germany; 7 Research Unit Apoptosis in Hematopoietic Stem Cells, Helmholtz Center Munich, 81377 Munich, Germany; 8 Cancer & Immunometabolism Research Group, Gene Center, LMU Munich, 81377 Munich, Germany

**Keywords:** Acute myeloid leukaemia, Cancer metabolism, Cancer genetics

## Abstract

ZBTB7A is frequently mutated in acute myeloid leukemia (AML) with t(8;21) translocation. However, the oncogenic collaboration between mutated ZBTB7A and the RUNX1–RUNX1T1 fusion gene in AML t(8;21) remains unclear. Here, we investigate the role of ZBTB7A and its mutations in the context of normal and malignant hematopoiesis. We demonstrate that clinically relevant ZBTB7A mutations in AML t(8;21) lead to loss of function and result in perturbed myeloid differentiation with block of the granulocytic lineage in favor of monocytic commitment. In addition, loss of ZBTB7A increases glycolysis and hence sensitizes leukemic blasts to metabolic inhibition with 2-deoxy-d-glucose. We observed that ectopic expression of wild-type ZBTB7A prevents RUNX1-RUNX1T1-mediated clonal expansion of human CD34+ cells, whereas the outgrowth of progenitors is enabled by ZBTB7A mutation. Finally, ZBTB7A expression in t(8;21) cells lead to a cell cycle arrest that could be mimicked by inhibition of glycolysis. Our findings suggest that loss of ZBTB7A may facilitate the onset of AML t(8;21), and that RUNX1-RUNX1T1-rearranged leukemia might be treated with glycolytic inhibitors.

## Introduction

Recently, we and others found the transcription factor ZBTB7A mutated in acute myeloid leukemia (AML) with translocation t(8;21), at frequencies ranging from 9.4 to 23% [1–6]. Hotspot mutations result either in loss (A175fs) or alteration (R402) of the C-terminal zinc finger domain, which is critical for DNA-binding of ZBTB7A [1]. The specific association of *ZBTB7A* alterations with the t(8;21) subgroup of AML patients points toward a unique mechanism of leukemogenesis. While the RUNX1–RUNX1T1 fusion gene, which results from the t(8;21) translocation, has been studied extensively, it remains unclear how it may provide a fertile ground for the acquisition of genetic lesions in ZBTB7A.

This oncogenic collaboration may arise from a complementary action on perturbed hematopoietic development (i.e., block of specific arms of the myeloid lineage). Expression of full length RUNX1–RUNX1T1 in a murine model does not cause leukemia [7, 8], but causes a partial block of myeloid differentiation with suppression of erythropoiesis and accumulation of immature granulocytes [9]. Interestingly, Zbtb7a has been described as a key regulator of hematopoietic differentiation with an essential role in erythropoiesis [10], lineage choice of B vs T lymphopoiesis [11] and long-term stem cell maintenance [12]. The involvement of ZBTB7A in myeloid differentiation has so far not been completely clarified, although *Zbtb7a* null mouse studies showed a deficiency of mature myeloid cells in fetal liver [12]. This suggests that *ZBTB7A* mutation could lead to a block of terminal myeloid differentiation, collaborating with RUNX1–RUNX1T1 to produce a complete differentiation block.

Another way in which ZBTB7A mutation may collaborate with RUNX1–RUNX1T1 is related to growth regulation and metabolism. While expression of RUNX1–RUNX1T1 in stem cells causes increased proliferation [13], expression in myeloid cell lines results in growth arrest. This growth arrest is related to downregulation of *MYC* [14] and *PKM2* [15]—a master regulator of glycolysis and a key enzyme of the glycolytic pathway, respectively. Moreover, AML t(8;21) has been described to depend on glycolytic metabolism for its survival [16]. In turn, ZBTB7A can directly repress the transcription of several genes implicated in glycolysis (*SLC2A3*, *PFKP,* and *PKM)* in an *MYC*-independent manner, and ZBTB7A knockdown in a colon cancer cell line resulted in increased glycolysis and proliferation [17]. ZBTB7A function in glycolysis regulation has so far not been studied extensively in the hematopoietic system, but the observed upregulation of glycolytic genes upon ZBTB7A mutation in our patient cohort [1] may counteract the growth arrest caused by RUNX1–RUNX1T1 in AML t(8;21).

Considering that ZBTB7A plays a critical role both in regulation of differentiation and cellular growth, alteration in either of the two functions may contribute to RUNX1-RUNX1T1-dependent leukemogenesis. In the present study, we investigate the effect of ZBTB7A mutation on cellular differentiation, glycolysis regulation, and RUNX1–RUNX1T1 directed cell expansion.

## Results

### ZBTB7A promotes granulopoiesis while blocking monocytic differentiation

**Fig. 1:**
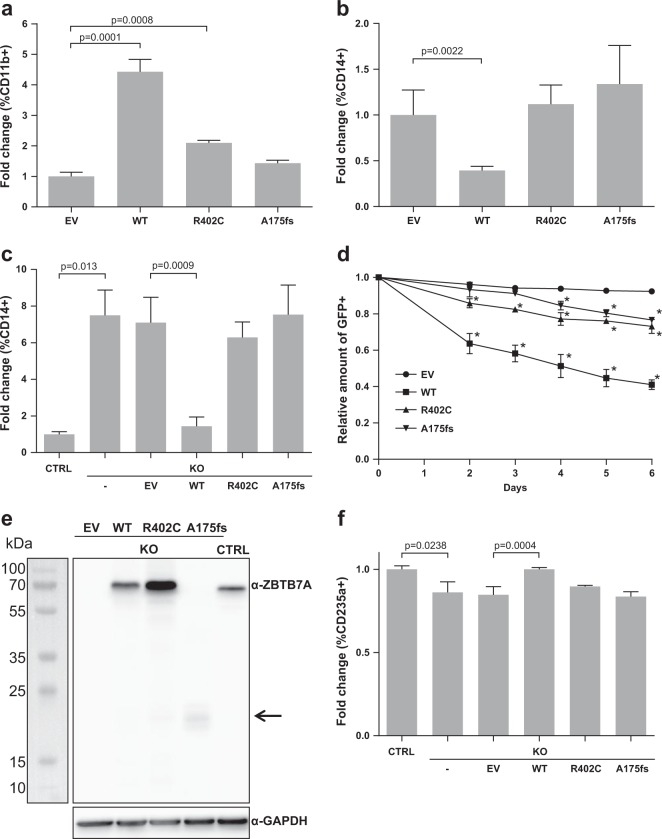
ZBTB7A promotes granulopoiesis while blocking monocytic differentiation. **a** HL60 cells stably expressing an empty vector (EV), ZBTB7A WT or mutants were differentiated by ATRA treatment. CD11b expression was assessed by flow cytometry. **b** HL60 cells stably expressing ZBTB7A WT or mutants were differentiated by PMA treatment. CD14 expression was assessed by flow cytometry. **c** HL60 ZBTB7A KO and HL60 ZBTB7A KO stably expressing ZBTB7A WT or mutants without induction of differentiation. CD14 expression was assessed by flow cytometry. **d** Competitive growth of HL60 cells stably expressing ZBTB7A WT or mutants. **e** Western blot from K562 cells, arrow indicates low levels of the ZBTB7A A175fs mutant. **f** K562 ZBTB7A KO without induction of differentiation. CD235a expression was assessed by flow cytometry. *p value < 0.05 compared with control cells.

Since ZBTB7A is a key regulator of hematopoietic linage fate decisions, we set out to compare the effect of ZBTB7A wild type (WT) and mutants in the context of myeloid differentiation. The cell line HL60 is a well-established model for granulocytic and monocytic differentiation [18, 19]. Therefore, we generated HL60 cells stably expressing ZBTB7A WT or mutants. Granulocytic differentiation induced by all-trans retinoic acid (ATRA) was increased by ZBTB7A WT, while this effect was significantly reduced for the mutants, with R402C showing residual activity (Fig. 1a, Supplementary Fig. 1a). In contrast, monocytic differentiation induced by phorbol 12-myristate 13-acetate (PMA) was reduced by ZBTB7A WT but not by the mutants (Fig. 1b, Supplementary Fig. 1b). In order to validate this effect, we generated an HL60 ZBTB7A knockout cell line. Interestingly, these cells presented a 5.5-fold increase in CD14 even without induction of differentiation (Fig. 1c, Supplementary Fig. 1c). Ectopic expression of ZBTB7A WT in the knockout cells restored CD14 to the native levels, while expression of the mutants had no effect (Fig. 1c, Supplementary Fig. 1d). With regard to potential therapeutic applications, we tested the PMA sensitivity of HL60 cells and found a significantly lower IC50 in absence of ZBTB7A (mean (pM): 256.6 in KO vs 619 in control; *p* value = 0.0002) (Supplementary Fig. 1e). We also observed that ZBTB7A WT expression lead to a loss of transduced cells in HL60 without cell sorting (Fig. 1d).

Since ZBTB7A was previously described to promote erythroid differentiation [10], we generated a K562 *ZBTB7A* knockout cell line (Fig. 1e). K562 cells can be used as a model for erythroid differentiation [20]. As expected, *ZBTB7A* knockout K562 cells presented a lower erythroid differentiation (13.89 ± 2.8% reduction, *p* value = 0.0238) when compared with control cells (Fig. 1f, Supplementary Fig. 1f). This impaired differentiation could be rescued by ectopic expression of ZBTB7A WT but not by the mutants (Fig. 1f, Supplementary Fig. 1g). These findings confirm the observation that R402C and A175fs result in loss of the regulatory function of ZBTB7A in myeloid differentiation.

### ZBTB7A blocks the differentiation of hematopoietic stem and progenitor cells (HSPCs)

**Fig. 2:**
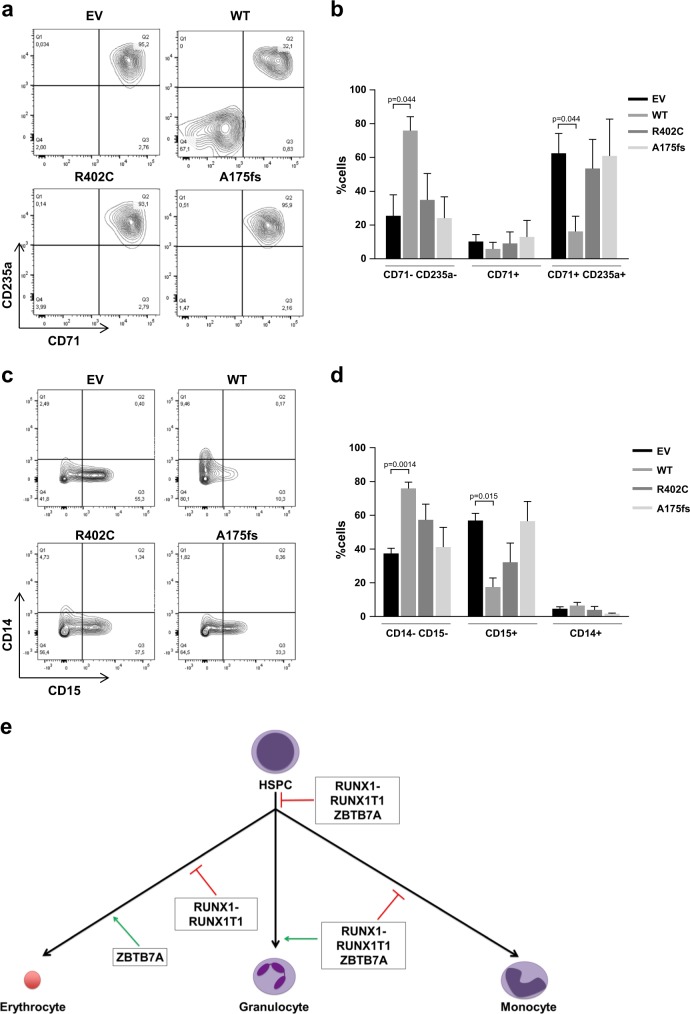
ZBTB7A blocks the differentiation of HSPC. Flow cytometry measurements of human bone marrow CD34+ cells stably expressing ZBTB7A WT or mutants are shown. **a** Representative results of cells primed for differentiation into erythrocytes using StemSpan Erythroid Expansion Supplement for 7 days. **b** Summary of three independent experiments. **c** Representative results of cells primed for differentiation into monocytes and granulocytes using HemaTox Myeloid Kit for 7 days. **d** Summary of three independent experiments. **e** Schematic representation of the effects of ZBTB7A and RUNX1–RUNX1T1 expression on hematopoietic linage commitment.

Considering that ZBTB7A was described to have a context-dependent effect on cell differentiation (i.e., block or promotion of differentiation) [21], we assessed the effect of ZBTB7A mutations on the HSPC compartment. To this aim, we generated human CD34+ cells stably expressing ZBTB7A WT or mutants. Upon differentiation, we observed a significant reduction of mature erythrocytes (CD71+ CD235a+) in WT expressing cells, consistent with previous reports [12]. In contrast, ZBTB7A mutant expressing cells differentiated to a similar extent as the control cells (Fig. 2a, b). When cells were differentiated to granulocytes and monocytes, we observed that WT transduced cells presented a reduction of CD15+ cells (corresponding to decreased granulopoiesis). Again, cells expressing the mutants did not exhibit this differentiation block (Fig. 2c, d). A schematic representation of the effects of ZBTB7A in differentiation is shown in Fig. 2e.

### Loss of ZBTB7A sensitizes to glycolysis inhibition

**Fig. 3:**
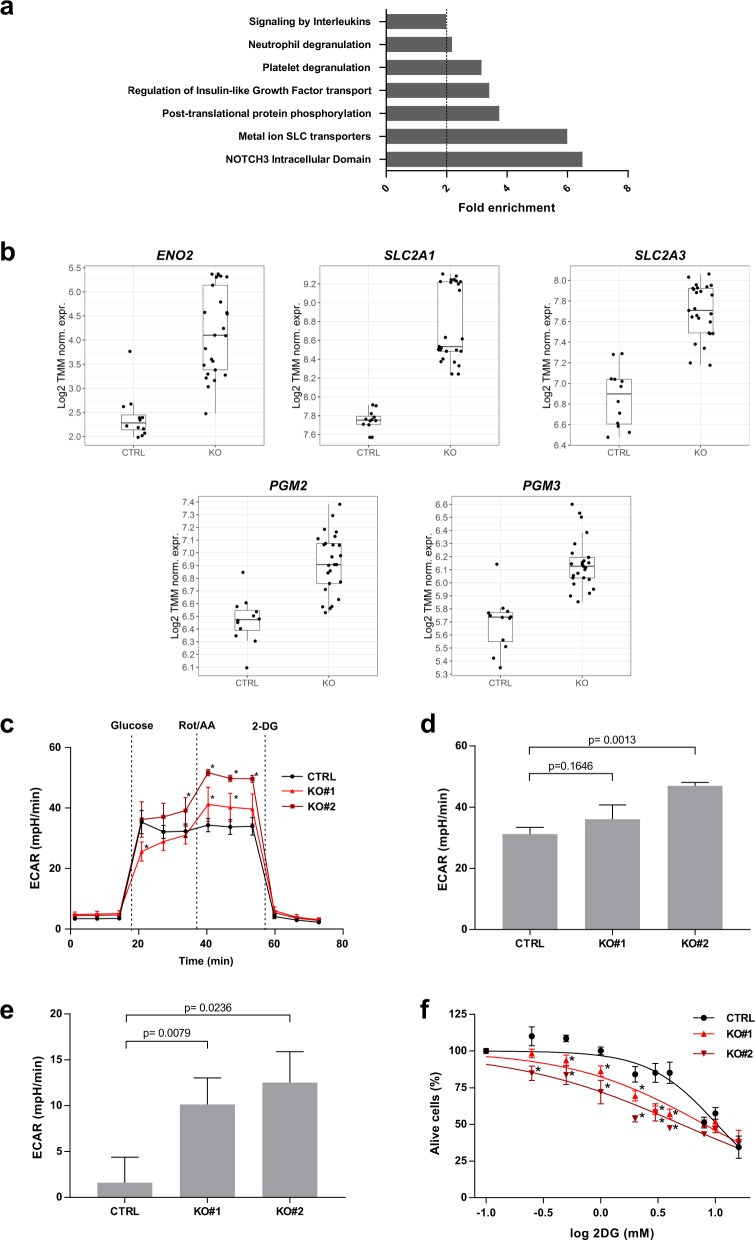
Loss of ZBTB7A sensitizes to glycolysis inhibition. K562 ZBTB7A KO and control cells underwent transcriptional profiling, metabolic flux analysis, and 2DG treatment. **a** Gene set enrichment analysis from RNA-Seq data. **b** Expression of *ENO2* (Enolase), *SLC2A1*, and *SLC2A3* (glucose membrane transporters), *PGM2* and *PGM3* (phosphoglycerate mutases) measured by RNA-Seq. **c** ECAR following the addition of glucose, Rot/AA, and 2DG. **d** Glycolytic capacity calculated as the ECAR after electron transport chain inhibition. **e** Glycolytic reserve calculated as the difference between glycolysis after glucose infusion and glycolytic capacity (F) cell viability after 2DG treatment. *p value < 0.05 compared with control cells.

In order to study the effects of ZBTB7A on metabolism described in other tissues [17], we analyzed transcriptomes by RNA-Seq in K562 ZBTB7A knockout and control clones (GSE140472). Differential expression analysis revealed 1089 genes deregulated between the two settings (adjusted *p* value < 0.05 and log-fold-change > 0.5) (Supplementary Fig. 2a). Gene set enrichment analysis revealed NOTCH3 transcriptional regulation as well as nutrient transport by solute carrier (SLC) proteins as the top significantly affected gene ontologies (Fig. 3a).

As ZBTB7A was previously described to be a negative regulator of glycolysis genes in colon cancer [17], we focused on the expression of genes implicated in glycolysis. This revealed an upregulation of the glucose transporters 1 (*SLC2A1*) and 3 (*SLC2A3*) in the knockout cells (Fig. 3b). Interestingly, two glycolytic enzymes not previously reported to be ZBTB7A targets were also found to be upregulated in KO cells: phosphoglycerate mutase isoforms 2 and 3 (*PGM2* and *PGM3*) responsible of converting 3-phosphoglycerate into 2-phosphoglycerate, and Enolase 2 (*ENO2*) responsible for converting 2-phospho-d-glycerate into phosphoenolpyruvate (Fig. 3b) (all comparisons *p* < 0.05). Other previously reported genes, such as *PKM* or *PKFP*, were not significantly deregulated in this setting (Supplementary Fig. 2b, c). The upregulated genes were confirmed as ZBTB7A targets (Supplementary Fig. 3) using publicly available ZBTB7A K562 ChIP-Seq data (ENCSR000BME, ENCODE database).

Based on these data, we selected two KO clones to test the functional impact of ZBTB7A loss on cellular metabolism. In metabolic flux analyses, ZBTB7A KO cells presented a slightly increased non-glycolytic acidification (Supplementary Fig. 4a, b). ZBTB7A KO cells did not show a statistically significant increase of glycolysis upon glucose administration (Fig. 3c, Supplementary Fig. 4c), but they presented a higher glycolytic capacity after inhibition of mitochondrial energy production compared with control (Fig. 3c–e). Interestingly, the increased energy demands of ZBTB7A KO cells could be compensated by the upregulation of mitochondrial respiration under glucose deprivation (Supplementary Fig. 4e). In addition, we observed that knockout cells were more sensitive to glycolysis inhibition with 2-deoxy-d-glucose (2DG) compared with control cells (mean IC50 (mM): 8.03 in KO#1 and 5.05 in KO#2 vs 10.34 in control; *p* values = 0.124 and 0.0005, respectively) (Fig. 3f). This effect was also confirmed by long-term treatment, where control cells were hardly affected by glycolysis inhibition, while knockout cells grew significantly slower (Supplementary Fig. 4d). The differences observed between the two KO clones tested may arise due to off-target effects of the Cas9 treatment or due to the fact that these lines were generated from single cells, amplifying any preexisting differences in the cell of origin. However, both clones show the same trends, namely, increased glycolysis after mitochondrial shutdown and increased sensitivity to glycolysis inhibition. In addition, we evaluated ex vivo sensitivity to 2DG in six different AML patient-derived xenografts (PDX) models where we could observe variable degrees of sensitivity (Supplementary Fig. 5).

### ZBTB7A prevents RUNX1-RUNX1T1-dependent clonal expansion

**Fig. 4:**
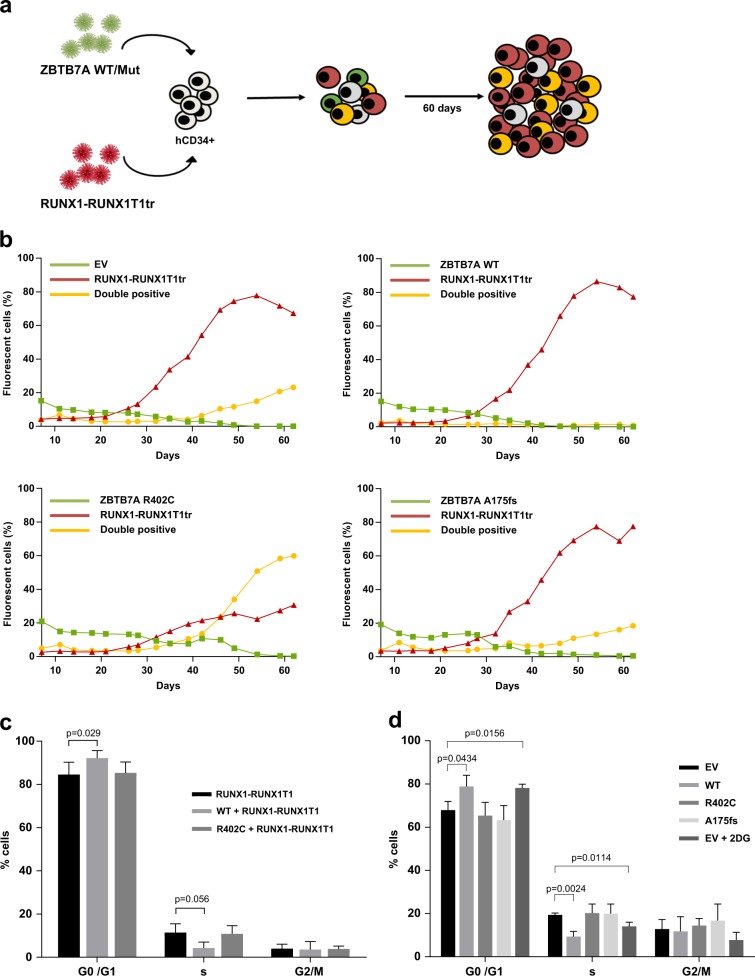
ZBTB7A prevents RUNX1-RUNX1T1tr-dependent clonal expansion of hCD34+ cells. **a** Schematic representation of the experimental layout. hCD34+ cells were transduced with *ZBTB7A* WT or mutants (GFP) and *RUNX1*–*RUNX1T1tr* (tomato). **b** FACS measurements of a representative competitive growth assay. The read out assessed was expansion or nonexpansion of GFP-tomato double positive cells. **c** Cell-cycle analysis of RUNX1–RUNX1T1 hCD34+ cells, transduced with the indicated constructs. **d** Cell-cycle analysis of GFP-positive Kasumi-1 cells stably expressing the indicated constructs.

Since ZBTB7A mutations are associated with AML t(8;21), we assessed the interplay between ZBTB7A and the RUNX1–RUNX1T1 fusion. To this aim, we used the truncated version of RUNX1–RUNX1T1 (hereafter referred to as RUNX1–RUNX1T1tr) that causes clonal expansion of hCD34+ cells [13]. A scheme of the experimental setting is provided in Fig. 4a. As expected, single positive cells expressing RUNX1–RUNX1T1tr expanded, while single positive cells expressing ZBTB7A WT or mutants did not (representative experiment in Fig. 4b, replicates of this experiment in Supplementary Fig. 6). Interestingly, cells expressing both RUNX1–RUNX1T1tr and ZBTB7A WT did not clonally expand and were quickly outcompeted by RUNX1–RUNX1T1tr single positive cells. The clonal expansion was enabled by the ZBTB7A mutations R402C and A175fs. Upon coexpression of these mutants, double positive cells expanded and no significant disadvantage over the RUNX1–RUNX1T1tr single positive cells was observed.

### ZBTB7A causes cell cycle arrest

In order to elucidate if the prevention of RUNX1-RUNX1T1tr-dependent clonal expansion by ZBTB7A arises either from downregulation of glycolysis, enhanced differentiation, or a combination of both effects, we expanded hCD34+ cells using RUNX1–RUNX1T1tr for 60 days and then transduced them with ZBTB7A WT and R402C. Cell-cycle analysis revealed that ZBTB7A WT expressing cells show a significant G0/G1 arrest in detriment of the S phase (Fig. 4c). In addition, we generated Kasumi-1 cells stably expressing ZBTB7A WT or mutants. We have previously shown that forced expression of ZBTB7A WT in Kasumi-1 causes a growth disadvantage [1]. Cell-cycle analysis revealed that ZBTB7A WT overexpressing cells show G0/G1 arrest in detriment of the S phase when compared with mutants and control (Fig. 4d). When glycolysis was inhibited by 2DG treatment, control cells showed a block of cell-cycle progression, reminiscent of the effect caused by ZBTB7A WT overexpression (Fig. 4d). At the same time, differentiation marker analysis (CD11b, CD14, and CD15) did not show any significant difference between the conditions tested (Supplementary Fig. 7).

## Discussion

*ZBTB7A* mutations in the context of AML have not yet been extensively characterized. In the present study, we demonstrated that ZBTB7A mutations have a loss-of-function phenotype with regard to differentiation and cell-cycle regulation (Figs. 1a, b, 2 and 4c, d). Moreover, ZBTB7A KO effects were only rescued by ectopic expression of the WT form but not by the mutants (Fig. 1c, f). We could though observe a slight residual activity of the point mutant R402C (Fig. 1a), as already described in other readouts [1]. Despite a previous report in the context of colon cancer suggesting that ZBTB7A zinc finger mutations act in a dominant negative manner [22], we could not find any evidence for this effect in our models, even when the expression of the R402C mutant was slightly higher than the control (Fig. 1e). The fact that the mutation A175fs results in an unstable truncated protein (Fig. 1e) also argues for a loss-of-function mechanism. In addition, we showed that the previously reported antiproliferative effect of ZBTB7A [1] is not exclusive to the t(8;21) background, as shown by loss of ZBTB7A WT expressing HL60 cells (Fig. 1d). This result is consistent with the assumption that ZBTB7A acts as a tumor suppressor and with our observation that higher *ZBTB7A* expression levels correlate with longer survival in cytogenetically normal AML patients [1]. Our results also corroborate a previously described role of ZBTB7A in erythroid differentiation (Figs. 1f, 2a, b) [10, 12] and suggest that ZBTB7A can block myeloid differentiation of HSPC (Fig. 2c, d).

The most puzzling fact about ZBTB7A mutations in AML is their exclusive presence in the context of core binding factor leukemia, mainly in t(8;21) AML [1–6], which suggests a specific collaboration between RUNX1–RUNX1T1 and loss of ZBTB7A function. Of note, it was previously reported that RUNX1–RUNX1T1 causes a block of the monocytic and erythrocytic linages in favor of granulocytic differentiation in mouse and zebrafish [9, 23]. In addition, an accumulation of neutrophils in the bone marrow of mice was observed [24]. All these models failed to present any leukemic disease. Interestingly, our HL60 model indicates that ZBTB7A has a role in directing cells into the granulocytic compartment while blocking monocytic differentiation (Fig. 1a–c). A loss of ZBTB7A function may therefore increase the block of myeloid differentiation initiated by t(8;21) (Fig. 2e). These results are in contrast to other reports based on cell lines stating that RUNX1–RUNX1T1 is sufficient to completely block granulocytic differentiation [25], however, such effect likely depends on the cellular context. Furthermore, AML t(8;21) was described to depend on glycolysis for its survival, specifically depending on PFKP and SLC2A3 [16], both direct targets of ZBTB7A [17]. This is further supported by the fact that the t(8;21) translocation positive Kasumi-1 cell line is highly sensitive to glycolysis inhibition [26]. In this study, we show that loss of ZBTB7A increases the expression of *SLC* glucose transporter genes as well as *ENO2, PGM2*, and *PGM3* (Fig. 3b), increasing glycolysis (Fig. 3c) and sensitizing to glycolysis inhibition (Fig. 3f). Interestingly, inhibition of mitochondrial respiration demasked a profoundly increased glycolytic reserve in ZBTB7A KO cells (Fig. 3e). This observation may encourage further studies regarding a possible advantage for ZBTB7A mutant cells in hypoxic environments. In vitro treatment of PDX cells revealed different degrees of sensitivity to 2DG (Supplementary Fig. 5), suggesting that response might be variable between patients and may depend on the genetic context. We also observed interference in RUNX1-RUNX1T1tr-dependent outgrowth of hCD34+ cells by forced ZBTB7A expression (Fig. 4b, Supplementary Fig. 6). Expression of ZBTB7A WT in a t(8;21) rearranged background does not cause increased differentiation in comparison to mutants (Supplementary Fig. 7), however, it leads to a cell cycle arrest (Fig. 4c, d). This effect resembles the cell cycle arrest due to the inhibition of glycolysis through 2DG treatment (Fig. 4d). These observations indicate that ZBTB7A expression in t(8;21) leukemia may lead to a decreased glycolysis rate and cell cycle arrest, thus impairing leukemia development. While the translocation t(8;21) in AML was the first recurrent cytogenetic abnormality ever described in any cancer [27], a specific treatment for this entity is not yet available. This is in contrast to other leukemia-associated rearrangements, such as *PML-RARA* or *BCR-ABL1*, that are pharmacologically actionable [28, 29]. In the present study, we show that ZBTB7A can counteract RUNX1-RUNX1T1-dependent progenitor cell expansion through repression of glycolysis, opening up avenues for a targeted treatment of AML t(8;21) with metabolic inhibitors.

In summary, we have shown that ZBTB7A mutations contribute to a terminal block of myeloid differentiation as well as to deregulation of glycolysis. Further studies are required to elucidate the complex interplay between tumor metabolism and perturbed differentiation in myeloid malignancies.

## Methods

### Plasmids and cell culture

All cell lines were acquired from DSMZ (Braunschweig, Germany). HL60 and K562 were cultured in RPMI-1640 medium (Life Technologies, Darmstadt, Germany) with 10% fetal bovine serum (FBS) (Biochrom, Berlin, Germany) and 1% PenStrep (PAN-Biotech, Aidenbach, Germany). Kasumi-1 cells were cultured with RPMI-1640 medium, 1% PenStrep and 20% FBS.

Human bone marrow CD34+ cells, containing HSPCs, were purchased from Lonza (Cologne, Germany) and cultured using IMDM (GE Healthcare Life Sciences, Pasching, Austria) complemented with 20% FBS 2% glutamine, 100 U PenStrep, 20 ng/ml FLT3-l, 20 ng/ml GM-CSF, hIL-3 10 ng/ml, hIL-6 20 ng/ml, hSCF 20 ng/ml, hTPO 20 ng/ml all from Peprotech (Hamburg, Germany).

PDX were described before [30]. Briefly, cells were isolated from NOD.Cg-Prkdc^scid^ Il2rg^tm1Wjl^/SzJ (NSG) mice bone marrow and then cultured in StemPro-34 SFM Medium (StemCell Technologies, Grenoble, France) supplemented with 1% PenStrep, 1% L-Glutamine, 2% FBS and 10 ng/ml SCF, TPO and IL-3.

The pMSCV-IRES-GFP ZBTB7A WT, R402C, and A175fs were described before [1]. The pMSCV-RUNX1-RUNX1T1tr-IRES-tdTomato was described before [31]. pSpCas9(BB)-2A-GFP (px458) is available from Addgene (Plasmid #48138) and gRNA sequences targeting ZBTB7A (GACTCGAGGTACTCCTTGGCG or GCCGCCGCTGCCAGCTTCCCG) were cloned as described before [32].

### CRISPR/Cas9 knockout

K562 and HL60 cells were electroporated with px458 containing a gRNA targeting ZBTB7A or an empty vector using Lonza 2b electroporation system following the manufacturer’s recommendation. Single cells were sorted for GFP into a 96-well plate. Single cells were expanded and ZBTB7A status was assessed by Western blot and Sanger sequencing, respectively.

### Differentiation assays

Cells were transduced and sorted for GFP. Granulocytic differentiation of HL60 cells was induced with 2 µM ATRA treatment (Sigma-Aldrich, Taufkirchen, Germany) for 72 h followed by flow cytometry measurement of CD11b surface expression using a mouse PE-Cy7 anti-human CD11b antibody (clone: ICRF44, BD Biosciencies, Temse, Belgium). Monocytic differentiation of HL60 cells was induced with 0.5 nM PMA (Abcam, Cambridge, UK) treatment for 48 h followed by flow cytometry measurement of CD14 surface expression using a mouse PE-Cy7 anti-human CD14 antibody (clone: M5E2, BD Biosciencies). Erythroid differentiation of K562 was assessed by flow cytometry measurement of glycophorin A (CD235a) surface expression using a mouse PE anti-human glycophorin A antibody (clone: GA-R2, BD Biosciencies) without induction of differentiation.

A total of 5000 Human CD34+ bone marrow cells were seeded either in StemSpan SFEM with StemSpan Erythroid Expansion Supplement or HemaTox Myeloid Kit (StemCell Technologies) in a 96-well plate. Cells were incubated for 7 days and differentiation was assessed by flow cytometry. Erythroid differentiation potential was assessed as stated before and with an additional mouse APC anti-human CD71 antibody (clone: M-A712, BD Biosciencies). Granulocytic differentiation was assessed by a mouse APC anti-human CD15 antibody (clone: SSEA-1, Biolegend, London, UK) and monocytic differentiation as stated above.

### Metabolic flux analysis

In all, 8 × 10^4^ cells were plated with Seahorse XF RPMI medium, pH 7.4 in a XF96 cell culture microplate (both Agilent, Waghauesel-Wiesental, Germany) coated with Cell Tak (Corning, Berlin, Germany) according to the manufacturer’s instructions. Oxygen consumption rate (OCR) and extracellular acidification rate (ECAR) were measured at 37 °C using a Seahorse XFe96 Analyzer (Agilent). Three measurements of OCR and ECAR were taken before and after each sequential injection of glucose at a final concentration of 1 mM, rotenone/antimycin A (Rot/AA) at a final concentration of 0.5 µM and 2DG at a final concentration of 50 mM (all Agilent).

### Drug sensitivity assays

In all, 10^4^ cells were plated in a 96-well plate with increasing concentrations of 2DG or PMA in technical triplicates. Cells were incubated for 72 h and then viability was assessed using CellTiter-Blue Cell Viability Assay (Promega, Mannheim, Germany) following the recommended protocol.

### Human CD34+ cells competitive growth

Human CD34+ bone marrow cells were double transduced with constructs harboring ZBTB7A WT, R402C, or A175fs (marked with GFP) together with RUNX1–RUNX1T1tr (marked with tomato). Expansion of single and double fluorescent marker-positive cells was then followed by flow cytometry over 60 days after transduction as described before [31].

### Cell-cycle analysis

Kasumi-1 and hCD34 cells were transduced and 4 × 10^5^ cells harvested and resuspended in 500 µl PBS. DRAQ5 (Thermo Fisher Scientific, Darmstadt, Germany) was added at a final concentration of 5 µM and incubated for 15 min. Cells were then analyzed by flow cytometry gating for GFP-positive cells and single events.

### Statistical analysis

*P* values were calculated using two-tailed Student’s *t* test for single comparison and analysis of variance followed by Dunnett’s multiple comparisons test for multiple comparisons in GraphPad Prism 7.03 (GraphPad Software, Inc., San Diego, CA, USA). Similarity of variance was evaluated using the Brown–Forsythe test. Graphs show mean and standard deviation of the mean of three independent experiments unless stated otherwise. Asterisk indicates significant differences (*p* value < 0.05). Sample exclusion was not carried out. FACS results were analyzed with FlowJo v10 (FlowJo LLC, Ashland, OR, USA).

## Supplementary information

ZBTB7A prevents clonal expansion_Supplementary Information

## Data Availability

The RNA-Seq data from K562 ZBTB7A knockout cells supporting the findings of this study is available in the Gene Expression Omnibus repository, GEO accession: GSE140472.
