# Supplementary material for: ZBTB7A prevents RUNX1-RUNX1T1-dependent clonal expansion of human hematopoietic stem and progenitor cells
Source: Oncogene. 2020 Mar 2;39(15):3195–205. doi: 10.1038/s41388-020-1209-4 (PMC7142018; doi:10.1038/s41388-020-1209-4)
Supplement: Supplementary file 1 — ZBTB7A prevents clonal expansion_Supplementary Information [file 41388_2020_1209_MOESM1_ESM.pptx]

## Slide 1
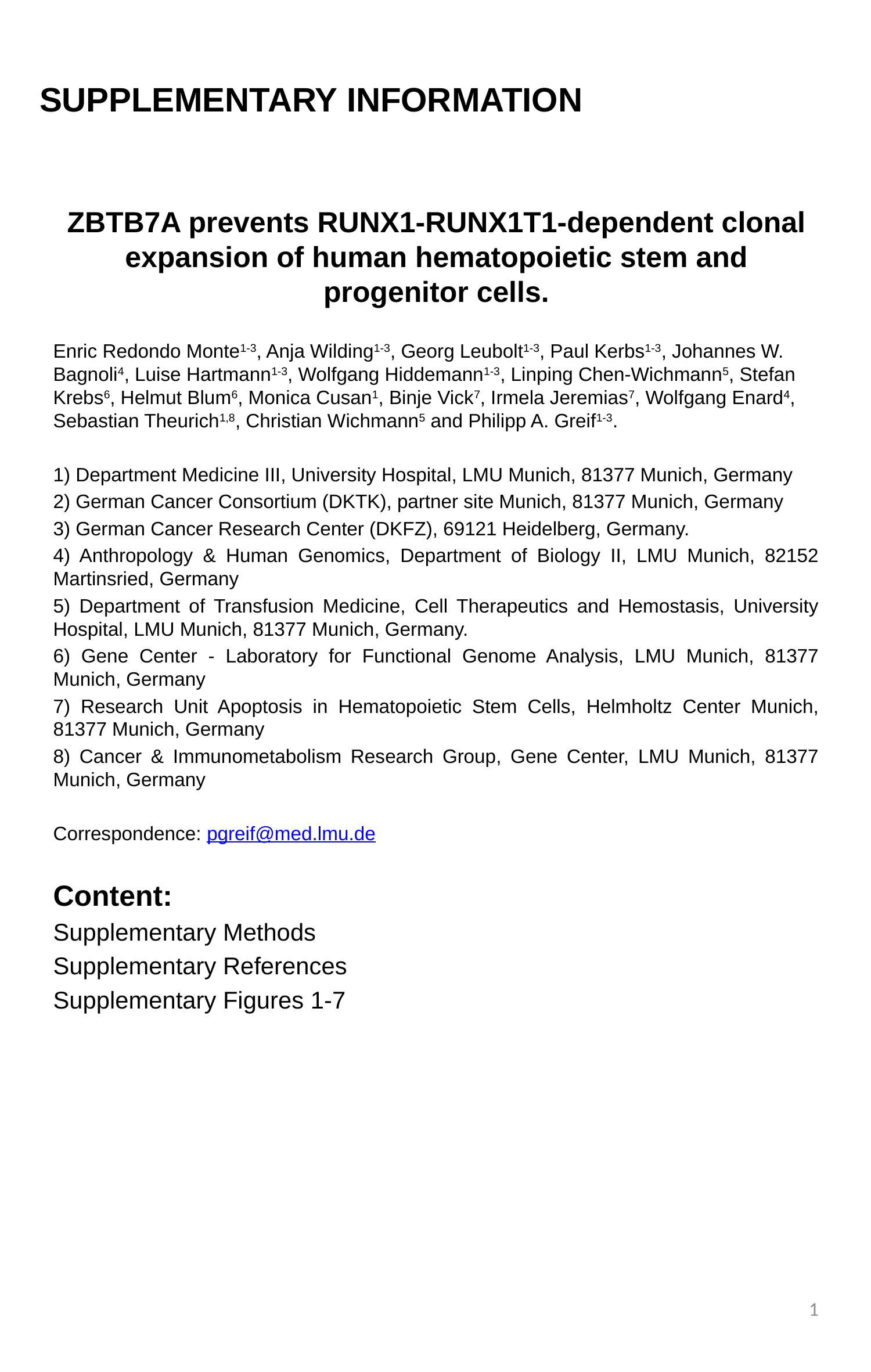

# SUPPLEMENTARY INFORMATION
ZBTB7A prevents RUNX1-RUNX1T1-dependent clonal expansion of human hematopoietic stem and progenitor cells.
Enric Redondo Monte1-3, Anja Wilding1-3, Georg Leubolt1-3, Paul Kerbs1-3, Johannes W. Bagnoli4, Luise Hartmann1-3, Wolfgang Hiddemann1-3, Linping Chen-Wichmann5, Stefan Krebs6, Helmut Blum6, Monica Cusan1, Binje Vick7, Irmela Jeremias7, Wolfgang Enard4, Sebastian Theurich1,8, Christian Wichmann5 and Philipp A. Greif1-3.
1) Department Medicine III, University Hospital, LMU Munich, 81377 Munich, Germany
2) German Cancer Consortium (DKTK), partner site Munich, 81377 Munich, Germany
3) German Cancer Research Center (DKFZ), 69121 Heidelberg, Germany.
4) Anthropology & Human Genomics, Department of Biology II, LMU Munich, 82152 Martinsried, Germany
5) Department of Transfusion Medicine, Cell Therapeutics and Hemostasis, University Hospital, LMU Munich, 81377 Munich, Germany.
6) Gene Center - Laboratory for Functional Genome Analysis, LMU Munich, 81377 Munich, Germany
7) Research Unit Apoptosis in Hematopoietic Stem Cells, Helmholtz Center Munich, 81377 Munich, Germany
8) Cancer & Immunometabolism Research Group, Gene Center, LMU Munich, 81377 Munich, Germany
Correspondence: pgreif@med.lmu.de
Content:
Supplementary Methods
Supplementary References
Supplementary Figures 1-7
1

## Slide 2
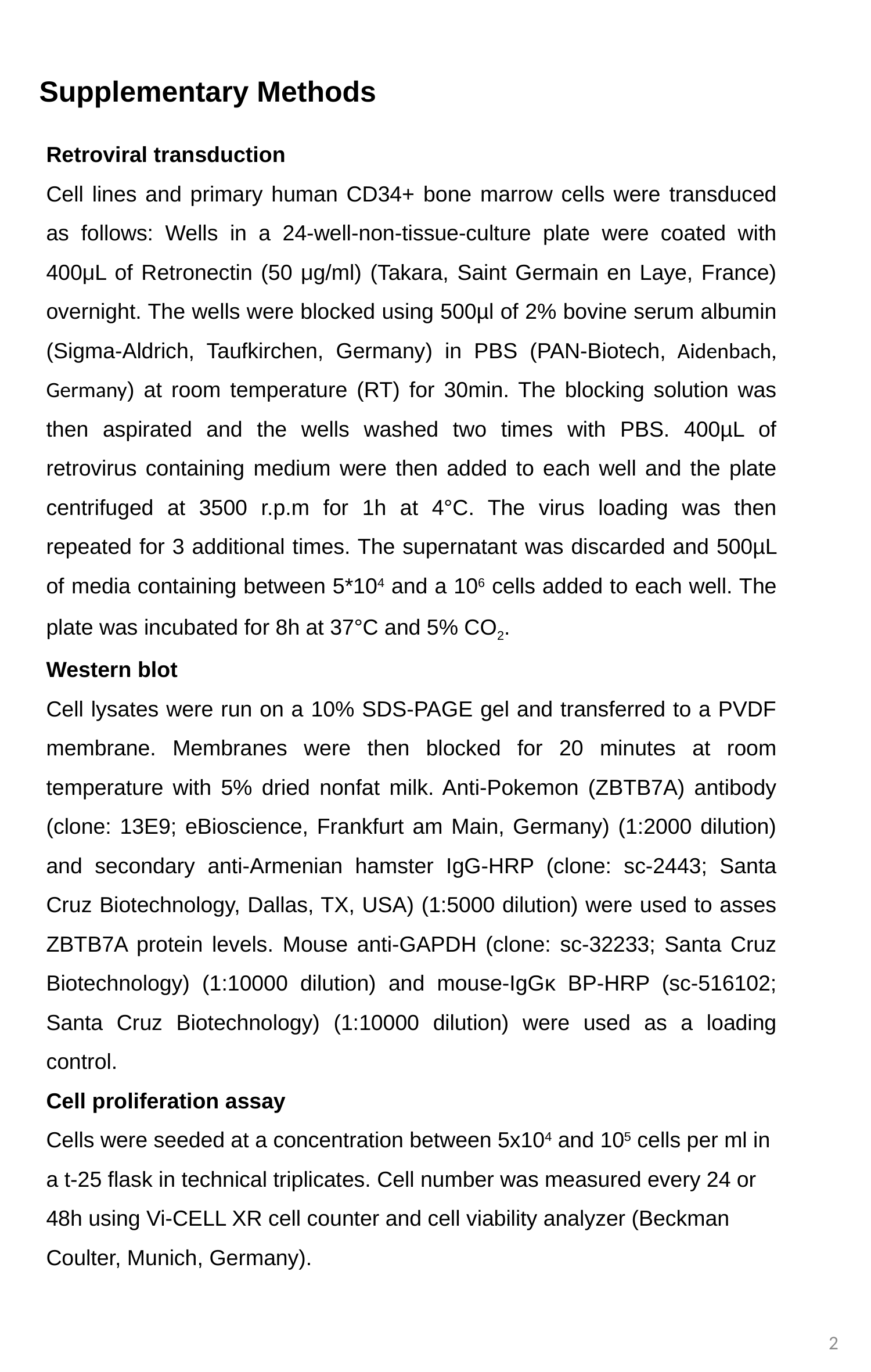

Supplementary Methods
Retroviral transduction
Cell lines and primary human CD34+ bone marrow cells were transduced as follows: Wells in a 24-well-non-tissue-culture plate were coated with 400μL of Retronectin (50 μg/ml) (Takara, Saint Germain en Laye, France) overnight. The wells were blocked using 500µl of 2% bovine serum albumin (Sigma-Aldrich, Taufkirchen, Germany) in PBS (PAN-Biotech, Aidenbach, Germany) at room temperature (RT) for 30min. The blocking solution was then aspirated and the wells washed two times with PBS. 400µL of retrovirus containing medium were then added to each well and the plate centrifuged at 3500 r.p.m for 1h at 4°C. The virus loading was then repeated for 3 additional times. The supernatant was discarded and 500µL of media containing between 5*104 and a 106 cells added to each well. The plate was incubated for 8h at 37°C and 5% CO2.
Western blot
Cell lysates were run on a 10% SDS-PAGE gel and transferred to a PVDF membrane. Membranes were then blocked for 20 minutes at room temperature with 5% dried nonfat milk. Anti-Pokemon (ZBTB7A) antibody (clone: 13E9; eBioscience, Frankfurt am Main, Germany) (1:2000 dilution) and secondary anti-Armenian hamster IgG-HRP (clone: sc-2443; Santa Cruz Biotechnology, Dallas, TX, USA) (1:5000 dilution) were used to asses ZBTB7A protein levels. Mouse anti-GAPDH (clone: sc-32233; Santa Cruz Biotechnology) (1:10000 dilution) and mouse-IgGκ BP-HRP (sc-516102; Santa Cruz Biotechnology) (1:10000 dilution) were used as a loading control.
Cell proliferation assay
Cells were seeded at a concentration between 5x10^4^ and 105 cells per ml in a t-25 flask in technical triplicates. Cell number was measured every 24 or 48h using Vi-CELL XR cell counter and cell viability analyzer (Beckman Coulter, Munich, Germany).
2

## Slide 3
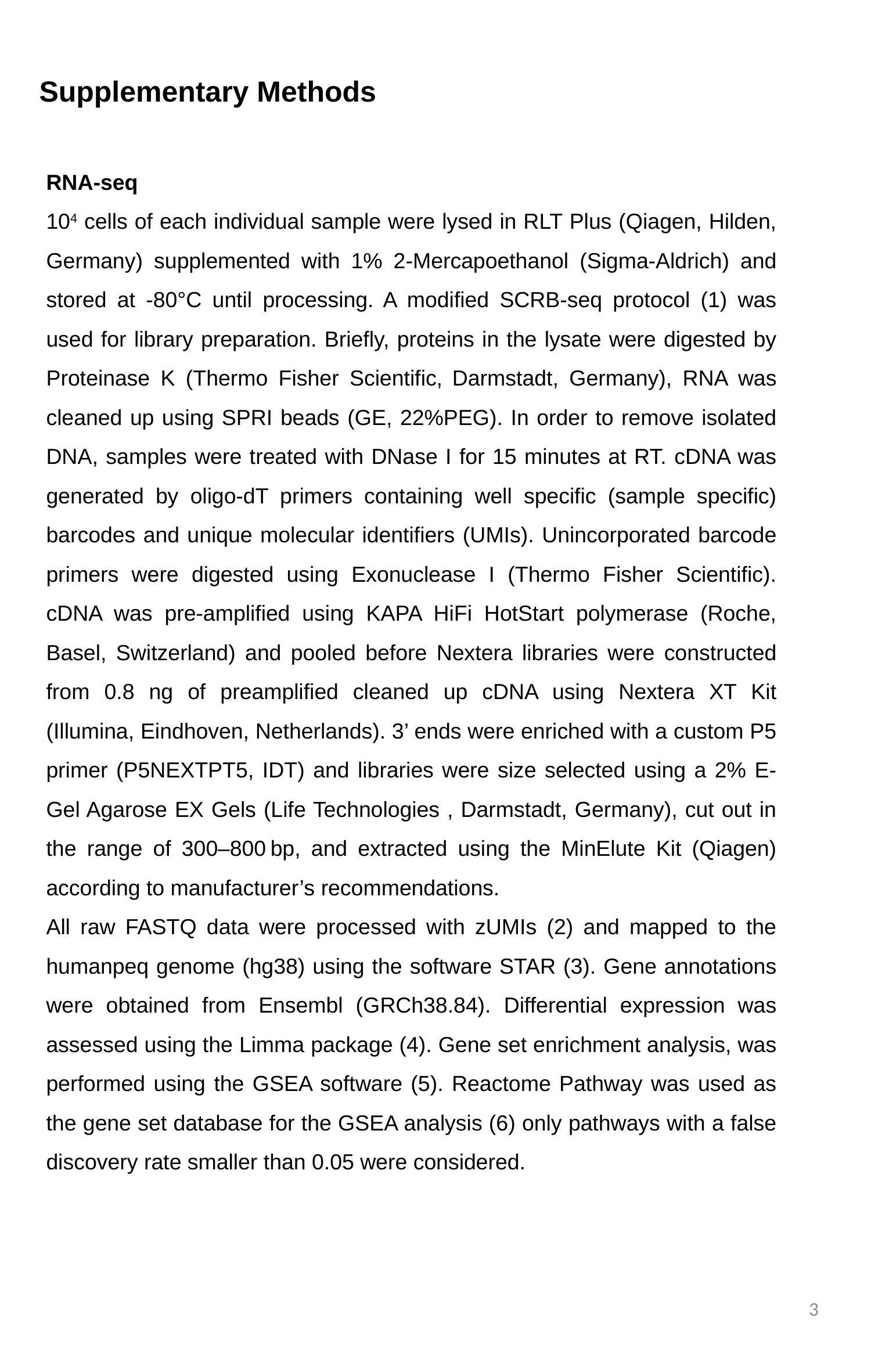

Supplementary Methods
RNA-seq
104 cells of each individual sample were lysed in RLT Plus (Qiagen, Hilden, Germany) supplemented with 1% 2-Mercapoethanol (Sigma-Aldrich) and stored at -80°C until processing. A modified SCRB-seq protocol (1) was used for library preparation. Briefly, proteins in the lysate were digested by Proteinase K (Thermo Fisher Scientific, Darmstadt, Germany), RNA was cleaned up using SPRI beads (GE, 22%PEG). In order to remove isolated DNA, samples were treated with DNase I for 15 minutes at RT. cDNA was generated by oligo-dT primers containing well specific (sample specific) barcodes and unique molecular identifiers (UMIs). Unincorporated barcode primers were digested using Exonuclease I (Thermo Fisher Scientific). cDNA was pre-amplified using KAPA HiFi HotStart polymerase (Roche, Basel, Switzerland) and pooled before Nextera libraries were constructed from 0.8 ng of preamplified cleaned up cDNA using Nextera XT Kit (Illumina, Eindhoven, Netherlands). 3’ ends were enriched with a custom P5 primer (P5NEXTPT5, IDT) and libraries were size selected using a 2% E-Gel Agarose EX Gels (Life Technologies , Darmstadt, Germany), cut out in the range of 300–800 bp, and extracted using the MinElute Kit (Qiagen) according to manufacturer’s recommendations.
All raw FASTQ data were processed with zUMIs (2) and mapped to the humanpeq genome (hg38) using the software STAR (3). Gene annotations were obtained from Ensembl (GRCh38.84). Differential expression was assessed using the Limma package (4). Gene set enrichment analysis, was performed using the GSEA software (5). Reactome Pathway was used as the gene set database for the GSEA analysis (6) only pathways with a false discovery rate smaller than 0.05 were considered.
3

## Slide 4
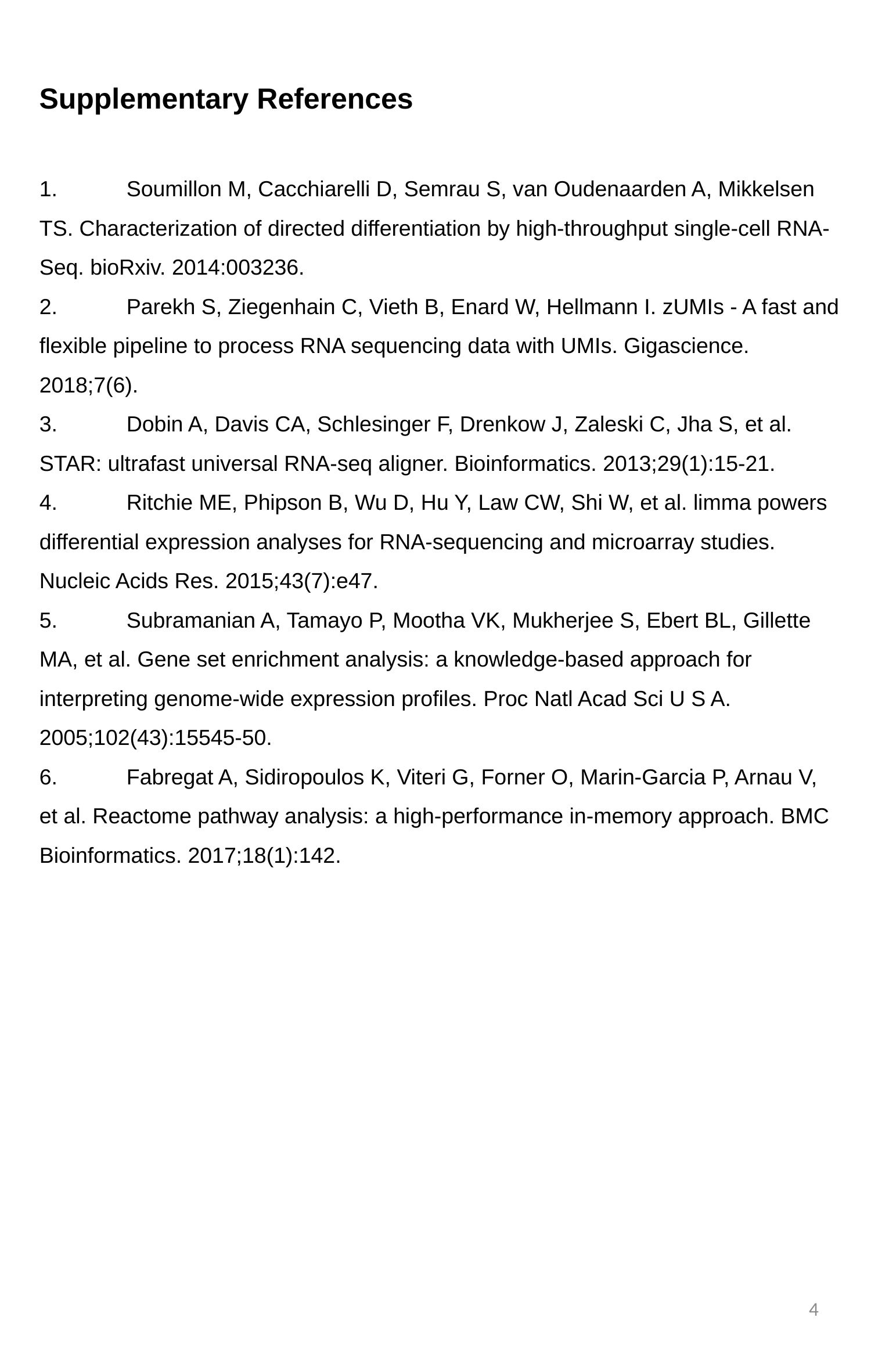

Supplementary References
1.	Soumillon M, Cacchiarelli D, Semrau S, van Oudenaarden A, Mikkelsen TS. Characterization of directed differentiation by high-throughput single-cell RNA-Seq. bioRxiv. 2014:003236.
2.	Parekh S, Ziegenhain C, Vieth B, Enard W, Hellmann I. zUMIs - A fast and flexible pipeline to process RNA sequencing data with UMIs. Gigascience. 2018;7(6).
3.	Dobin A, Davis CA, Schlesinger F, Drenkow J, Zaleski C, Jha S, et al. STAR: ultrafast universal RNA-seq aligner. Bioinformatics. 2013;29(1):15-21.
4.	Ritchie ME, Phipson B, Wu D, Hu Y, Law CW, Shi W, et al. limma powers differential expression analyses for RNA-sequencing and microarray studies. Nucleic Acids Res. 2015;43(7):e47.
5.	Subramanian A, Tamayo P, Mootha VK, Mukherjee S, Ebert BL, Gillette MA, et al. Gene set enrichment analysis: a knowledge-based approach for interpreting genome-wide expression profiles. Proc Natl Acad Sci U S A. 2005;102(43):15545-50.
6.	Fabregat A, Sidiropoulos K, Viteri G, Forner O, Marin-Garcia P, Arnau V, et al. Reactome pathway analysis: a high-performance in-memory approach. BMC Bioinformatics. 2017;18(1):142.
4

## Slide 5
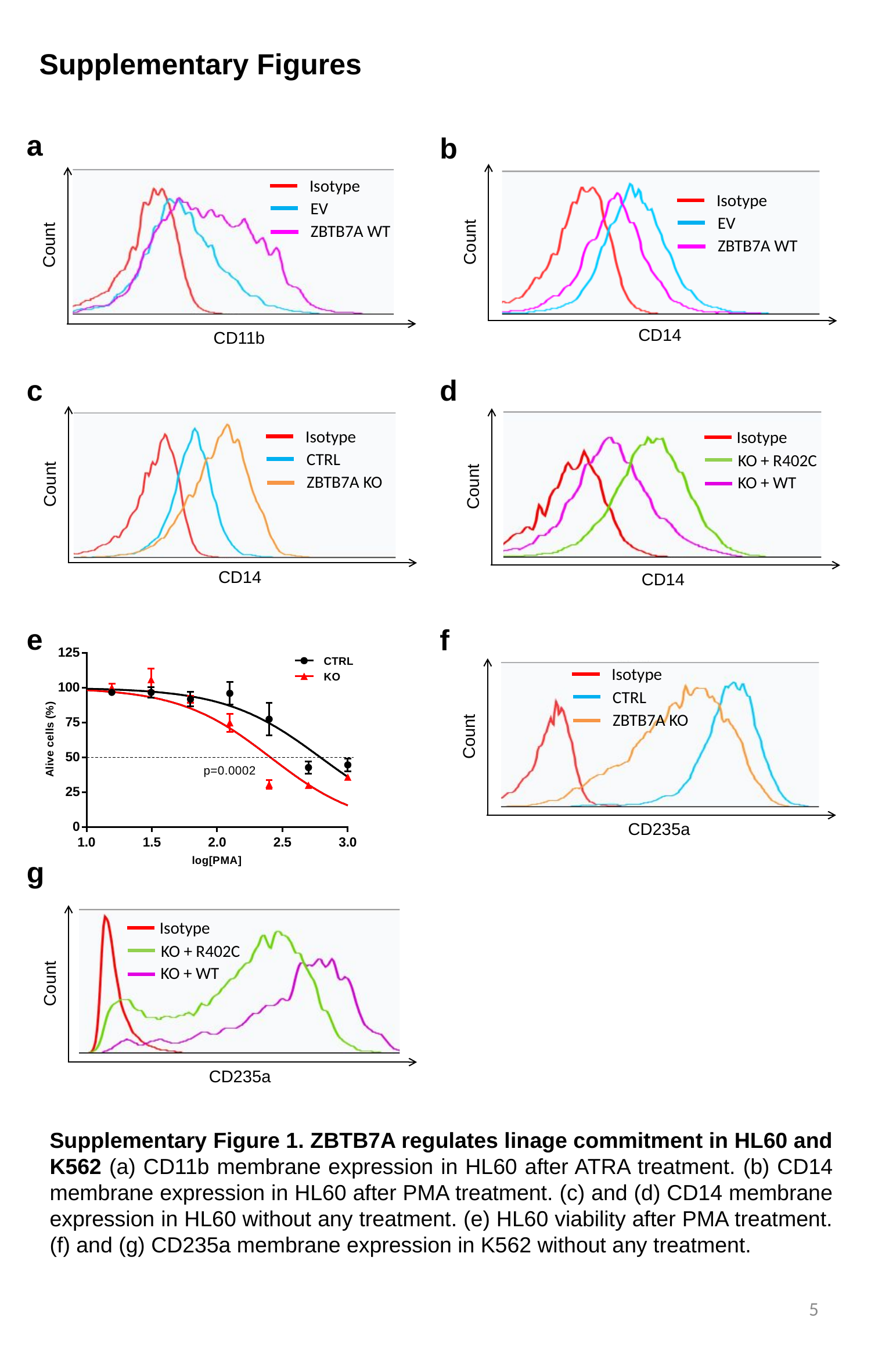

Supplementary Figures
a
b
Isotype
Isotype
EV
EV
ZBTB7A WT
Count
ZBTB7A WT
Count
CD14
CD11b
d
c
Isotype
Isotype
CTRL
KO + R402C
ZBTB7A KO
KO + WT
Count
Count
CD14
CD14
e
f
Isotype
CTRL
ZBTB7A KO
Count
CD235a
g
Isotype
KO + R402C
KO + WT
Count
CD235a
Supplementary Figure 1. ZBTB7A regulates linage commitment in HL60 and K562 (a) CD11b membrane expression in HL60 after ATRA treatment. (b) CD14 membrane expression in HL60 after PMA treatment. (c) and (d) CD14 membrane expression in HL60 without any treatment. (e) HL60 viability after PMA treatment. (f) and (g) CD235a membrane expression in K562 without any treatment.
5

## Slide 6
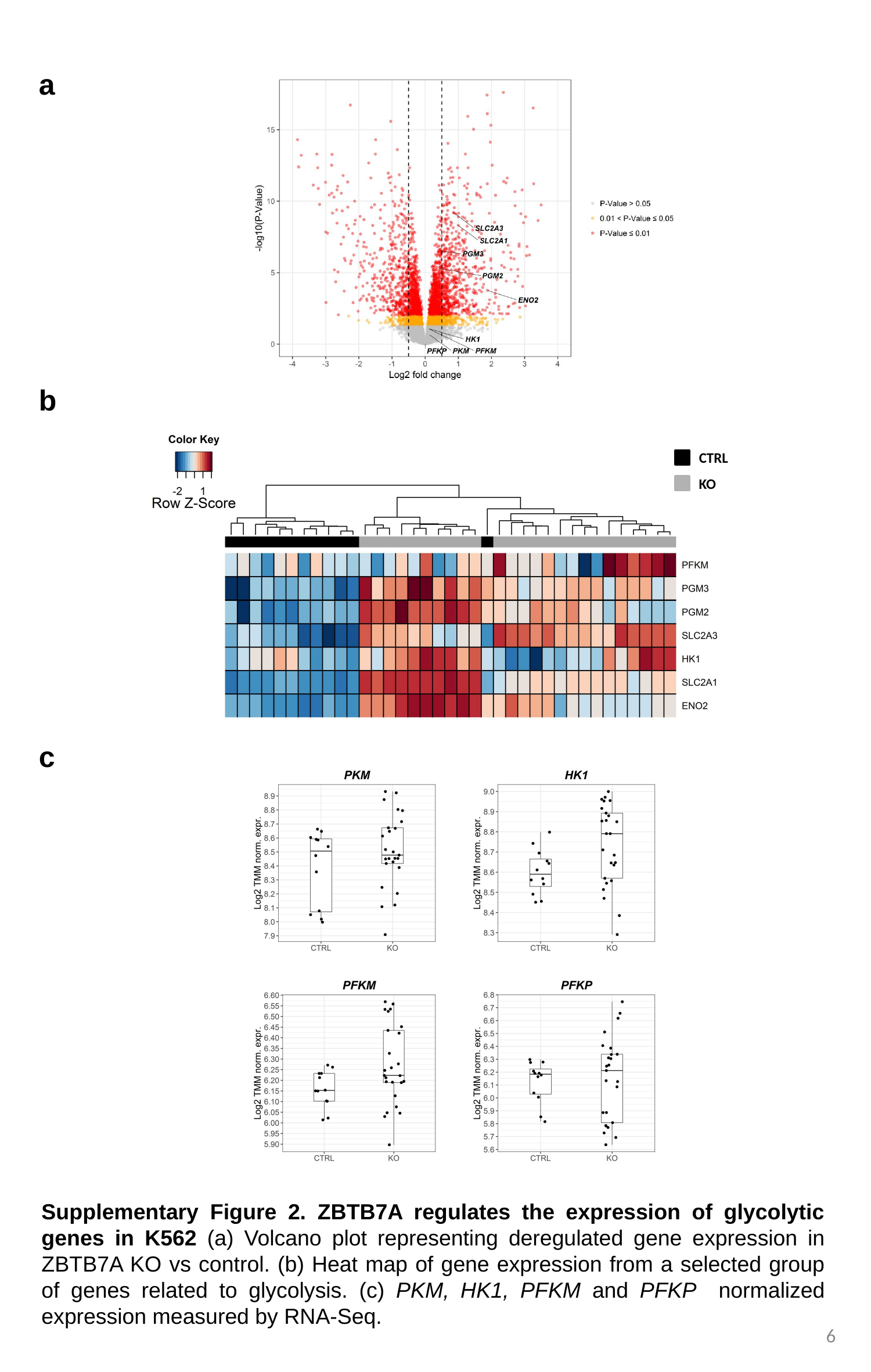

a
b
CTRL
KO
c
Supplementary Figure 2. ZBTB7A regulates the expression of glycolytic genes in K562 (a) Volcano plot representing deregulated gene expression in ZBTB7A KO vs control. (b) Heat map of gene expression from a selected group of genes related to glycolysis. (c) PKM, HK1, PFKM and PFKP normalized expression measured by RNA-Seq.
6

## Slide 7
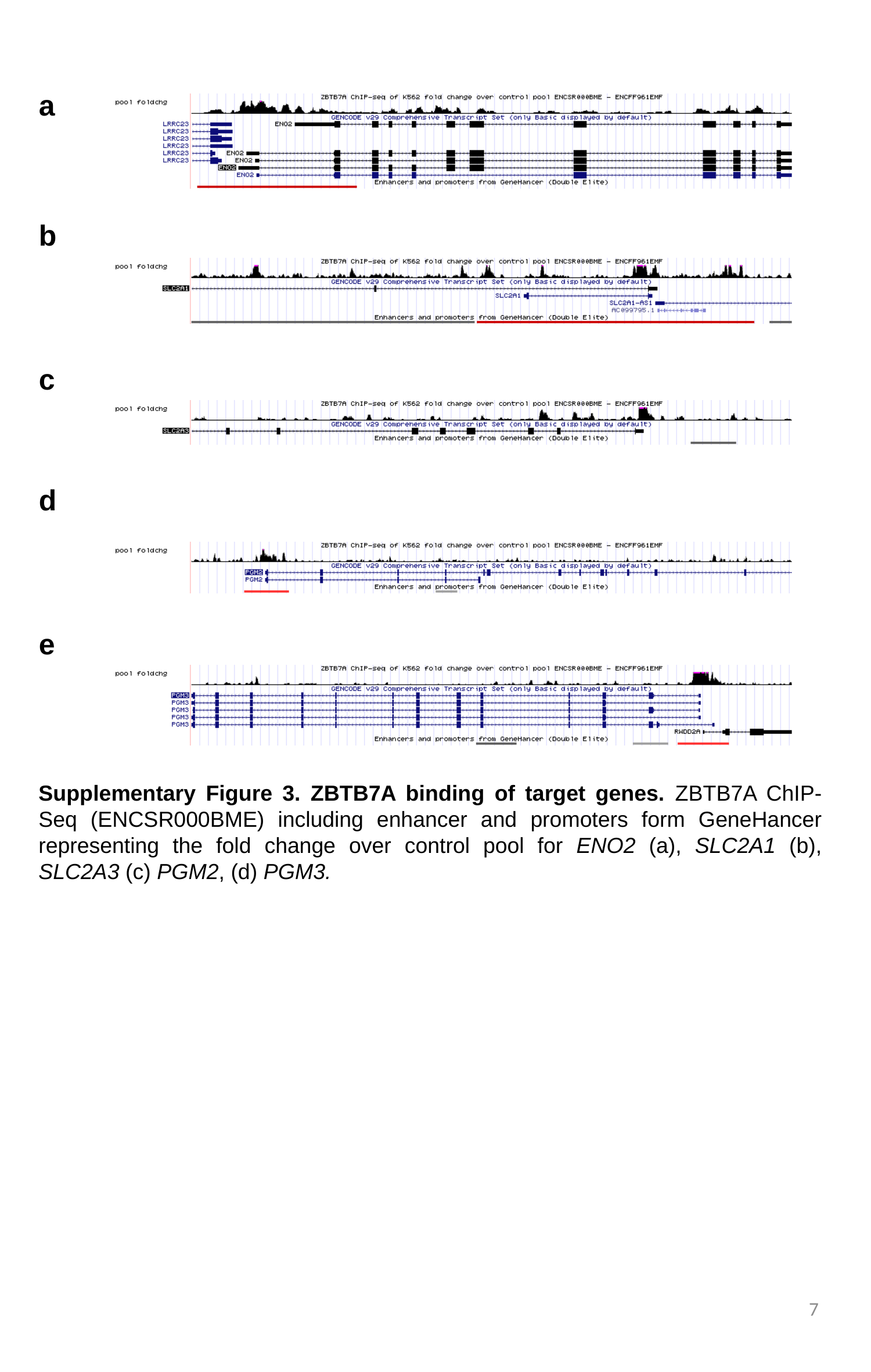

a
b
c
d
e
Supplementary Figure 3. ZBTB7A binding of target genes. ZBTB7A ChIP-Seq (ENCSR000BME) including enhancer and promoters form GeneHancer representing the fold change over control pool for ENO2 (a), SLC2A1 (b), SLC2A3 (c) PGM2, (d) PGM3.
7

## Slide 8
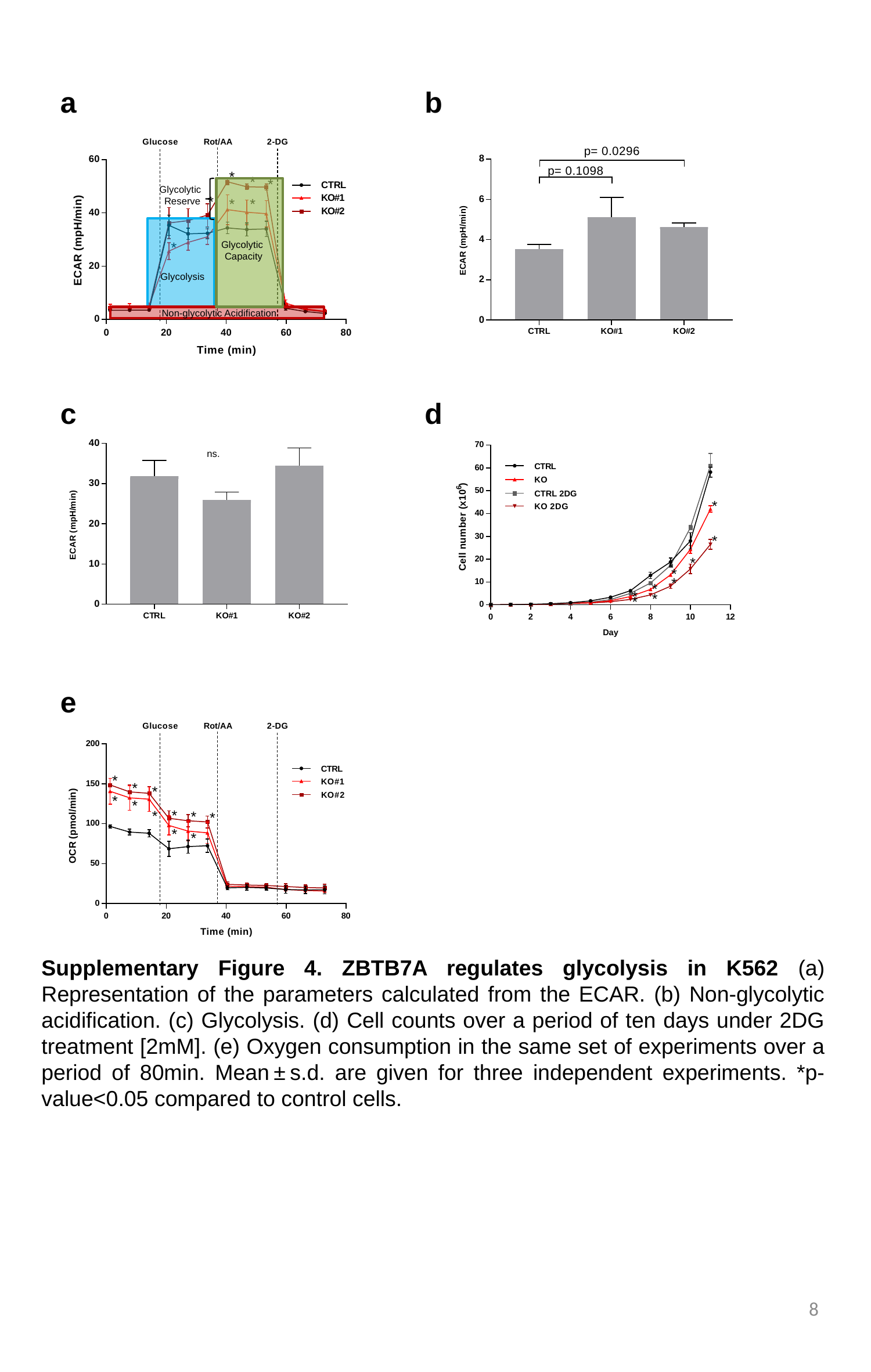

a
b
Glycolytic
Reserve
Glycolytic
Capacity
Glycolysis
Non-glycolytic Acidification
c
d
e
Supplementary Figure 4. ZBTB7A regulates glycolysis in K562 (a) Representation of the parameters calculated from the ECAR. (b) Non-glycolytic acidification. (c) Glycolysis. (d) Cell counts over a period of ten days under 2DG treatment [2mM]. (e) Oxygen consumption in the same set of experiments over a period of 80min. Mean ± s.d. are given for three independent experiments. *p-value<0.05 compared to control cells.
8

## Slide 9
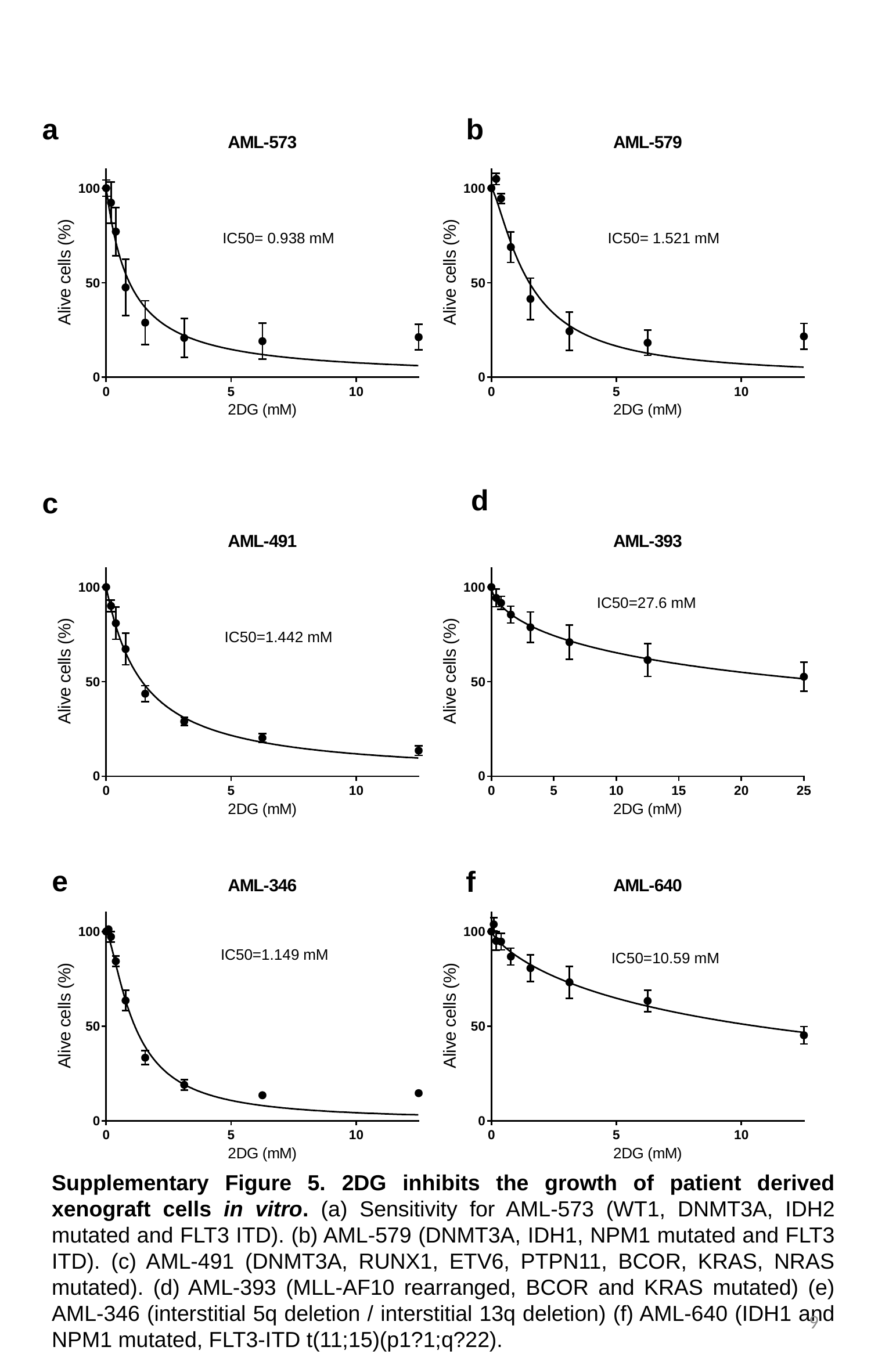

a
b
d
c
e
f
Supplementary Figure 5. 2DG inhibits the growth of patient derived xenograft cells in vitro. (a) Sensitivity for AML-573 (WT1, DNMT3A, IDH2 mutated and FLT3 ITD). (b) AML-579 (DNMT3A, IDH1, NPM1 mutated and FLT3 ITD). (c) AML-491 (DNMT3A, RUNX1, ETV6, PTPN11, BCOR, KRAS, NRAS mutated). (d) AML-393 (MLL-AF10 rearranged, BCOR and KRAS mutated) (e) AML-346 (interstitial 5q deletion / interstitial 13q deletion) (f) AML-640 (IDH1 and NPM1 mutated, FLT3-ITD t(11;15)(p1?1;q?22).
9

## Slide 10
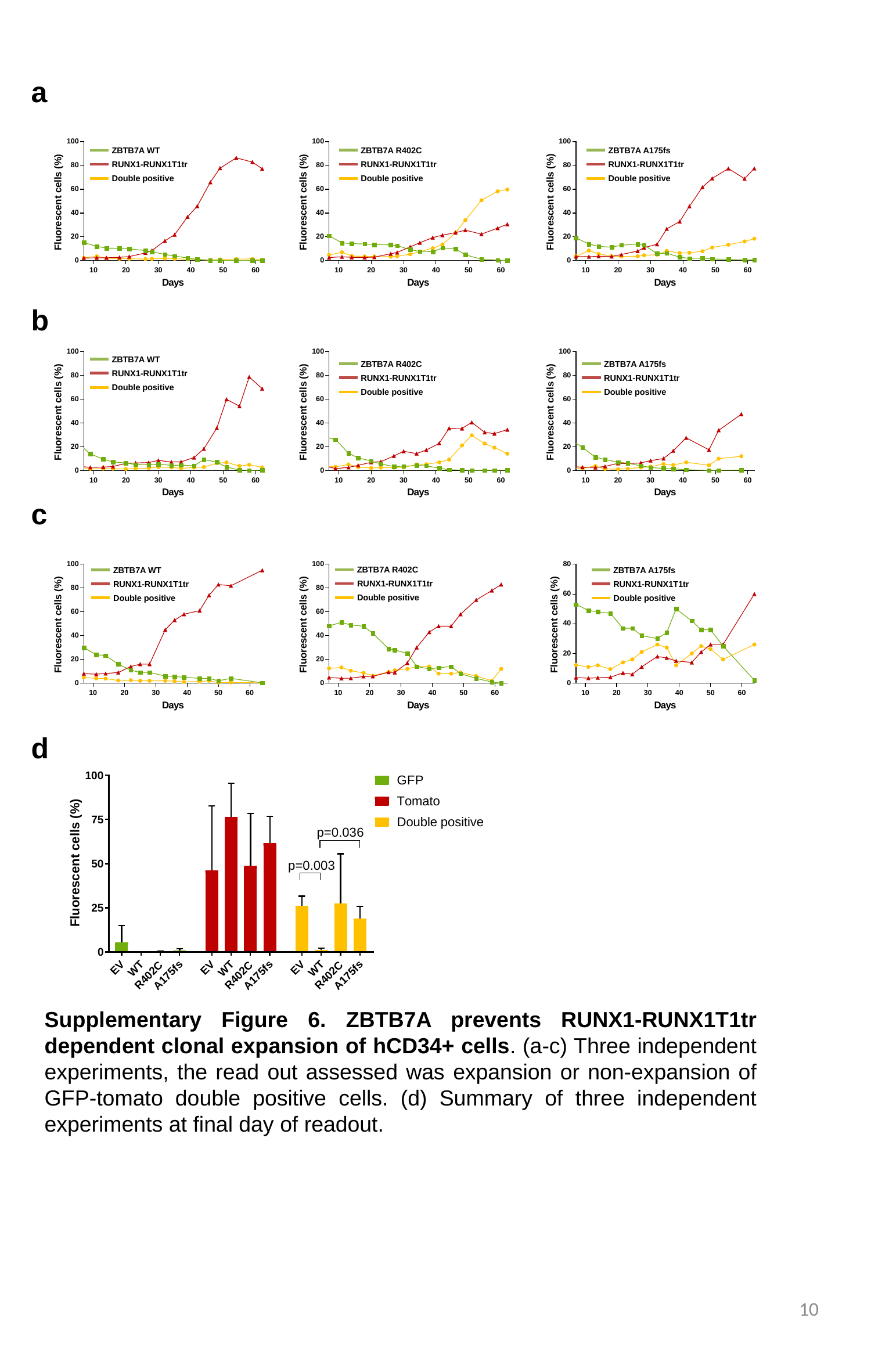

a
ZBTB7A R402C
RUNX1-RUNX1T1tr
Double positive
ZBTB7A A175fs
RUNX1-RUNX1T1tr
Double positive
ZBTB7A WT
RUNX1-RUNX1T1tr
Double positive
b
ZBTB7A WT
RUNX1-RUNX1T1tr
Double positive
ZBTB7A R402C
RUNX1-RUNX1T1tr
Double positive
ZBTB7A A175fs
RUNX1-RUNX1T1tr
Double positive
c
ZBTB7A R402C
RUNX1-RUNX1T1tr
Double positive
ZBTB7A WT
RUNX1-RUNX1T1tr
Double positive
ZBTB7A A175fs
RUNX1-RUNX1T1tr
Double positive
d
Supplementary Figure 6. ZBTB7A prevents RUNX1-RUNX1T1tr dependent clonal expansion of hCD34+ cells. (a-c) Three independent experiments, the read out assessed was expansion or non-expansion of GFP-tomato double positive cells. (d) Summary of three independent experiments at final day of readout.
10

## Slide 11
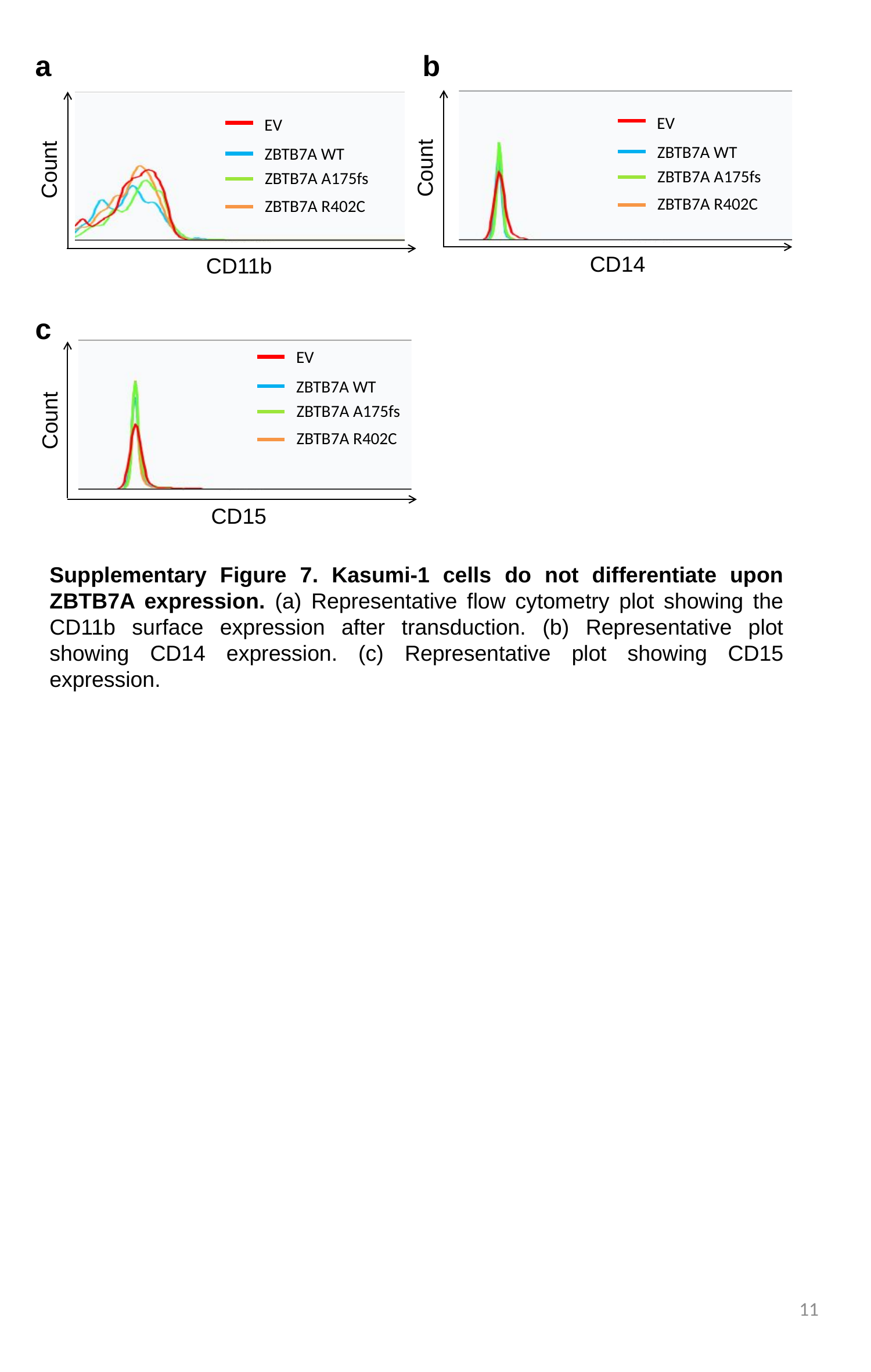

a
b
EV
EV
ZBTB7A WT
ZBTB7A WT
Count
Count
ZBTB7A A175fs
ZBTB7A A175fs
ZBTB7A R402C
ZBTB7A R402C
CD14
CD11b
c
EV
ZBTB7A WT
ZBTB7A A175fs
Count
ZBTB7A R402C
CD15
Supplementary Figure 7. Kasumi-1 cells do not differentiate upon ZBTB7A expression. (a) Representative flow cytometry plot showing the CD11b surface expression after transduction. (b) Representative plot showing CD14 expression. (c) Representative plot showing CD15 expression.
11
